# Histone chaperone‐mediated co‐expression assembly of tetrasomes and nucleosomes

**DOI:** 10.1002/2211-5463.13311

**Published:** 2021-10-19

**Authors:** Kei‐ichi Okimune, Shogo Hataya, Kazuki Matsumoto, Kanako Ushirogata, Petra Banko, Seiji Takeda, Taichi E. Takasuka

**Affiliations:** ^1^ Research Faculty of Agriculture Hokkaido University Sapporo Japan; ^2^ Graduate School of Global Food Resources Hokkaido University Sapporo Japan; ^3^ Faculty of Pharmaceutical Sciences Hokkaido University of Science Sapporo Japan; ^4^ Global Institute for Collaborative Research and Education Hokkaido University Sapporo Japan

**Keywords:** nucleosome, tetrasome, NAP1, wheat germ cell‐free synthesis, AFM

## Abstract

The nucleosome, a basic unit of chromatin found in all eukaryotes, is thought to be assembled through the orchestrated activity of several histone chaperones and chromatin assembly factors in a stepwise manner, proceeding from tetrasome assembly, to H2A/H2B deposition, and finally to formation of the mature nucleosome. In this study, we demonstrate chaperone‐mediated assembly of both tetrasomes and nucleosomes on the well‐defined Widom 601 positioning sequence using a co‐expression/reconstitution wheat germ cell‐free system. The purified tetrasomes and nucleosomes were positioned around the center of a given sequence. The heights and diameters were measured by atomic force microscopy. Together with the reported unmodified native histones produced by the wheat germ cell‐free platform, our method is expected to be useful for downstream applications in the field of chromatin research.

## Graphical abstract

In this study, we developed a novel histone chaperone‐mediated assembly method for tetrasomes and nucleosomes using a wheat germ cell‐free co‐expression system. *Drosophila* tetrasomes and nucleosomes were assembled on the Widom 601 sequence under near physiological conditions. Purified tetrasomes and nucleosomes were assessed by gel analyses and evaluated by atomic force microscopy.

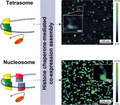

**Abbreviations**
AFM: atomic force microscopy
MNase: Micrococcal nuclease
NAP1: nucleosome assembly protein 1

In eukaryotic nuclei, genomic DNA is organized into chromatin. The basic building block of chromatin is the nucleosome, containing about 147 bp double‐stranded DNA wrapping around a histone octamer composed of two copies each of H2A, H2B, H3, and H4 in a left‐handed superhelix [1, 2, 3]. The open and closed chromatin higher order structures are dictated by local and genome‐wide assembly and disassembly of the nucleosome, which in turn regulate DNA replication, repair, gene expression, and others [4, 5, 6]. In the nuclei, the nucleosome is formed in a sequential process mediated by histone chaperones and chromatin assembly factors [7]. Two copies of the H3/H4 dimer are recruited to form an (H3/H4)_2_‐DNA complex (tetrasome), followed by deposition of H2A/H2B dimers [8]. The mechanisms and kinetics of nucleosome formation have been described, and the roles of several chromatin assembly factors have been studied in detail [9, 10]. Furthermore, it has been indicated that disassembly of H2A/H2B is actively proceeded during chromatin maintenance in the cell nucleus [11]. For instance, eviction of H2A/H2B from nucleosome could release the torsions created by the adjacent chromatin structure. One of the histone chaperones, nucleosome assembly protein 1 (NAP1), is known for its function in aiding nucleosome assembly at physiological ionic strength and is highly conserved among many species of eukaryotes [12, 13, 14, 15, 16, 17]. *In vitro* studies described the thermodynamics of NAP1‐mediated nucleosome assembly, and one of the important roles of NAP1 besides forming tetrasomes is to prevent non‐nucleosomal interaction of H2A/H2B dimers during nucleosome formation [9, 18]. These studies and other chromatin function‐related research essentially utilize recombinantly expressed, refolded, and preassembled core histones; thus, functions of chromatin‐associated proteins such as NAP1 during nucleosome formation under physiological conditions needs to be further tested.

Previously, we reported a novel *in vitro* chromatin reconstitution method using a wheat germ cell‐free co‐expression system, in which mRNAs encoding four core histones were co‐expressed with histone chaperones and ATP utilizing factors in the presence of a template circular DNA [19]. This method enables simultaneous histone synthesis and chromatin assembly under near physiological conditions *in vitro*. Additionally, the recent report from our group showed that the synthesized histones by a wheat germ cell‐free synthesis lacked any detectable posttranslational modification [20].

In this study, we sought to make our method more applicable for downstream applications to study chromatin assembly and disassembly dynamics under near physiological conditions. To this end, homogeneous *Drosophila melanogaster* tetrasomes and nucleosomes were assembled on the well‐defined Widom 601 nucleosome positioning sequence in a histone chaperone‐mediated manner [19, 21]. Furthermore, the affinity‐purified reconstituted tetrasomes and nucleosomes were analyzed by restriction enzyme digestion and micrococcal nuclease digestion, respectively. The typical heights and diameters of purified tetrasomes and nucleosomes were determined by atomic force microscopy. Overall, the current study provides evidence that our histone chaperone‐mediated co‐expression assembly of tetrasome and nucleosome enables preparation of a positioned homogeneous complex on the Widom 601 sequence, useful for downstream applications.

## Materials and methods

### Cloning

The DNA fragments of 185 bp and 307 bp containing the Widom 601 sequence at the center, 601_185 bp, and 601_307 bp, were amplified from pGEM‐3z/601 (Addgene, MA, USA) by PCR with the following primer pairs 5’‐GTCGCTGTTCAATACATGC‐3’ and 5’‐GACCCTATACGCGGCCGCC‐3’ for 601_185bp, and 5’‐TCGGTACCCGGGGATCCTC‐3’ and 5’‐CGGGATCCTAATGACCAAG‐3’ for 601_307bp. Both fragments were purified by phenol–chloroform‐IAA (25 : 24 : 1, v/v) extraction followed by ethanol precipitation and resuspended in 5 mm Tris/HCl buffer. The plasmid constructs harboring *Drosophila melanogaster* core histones and NAP1 were prepared as described previously [19]. In order to purify the reconstituted core histones‐DNA complexes, the histone H4 was designed to possess the N‐terminal His_6_‐tag, followed by a tobacco etch virus protease cleavage site [22]. The His_6_‐tag‐coding H4 gene was cloned into a pEU expression vector by a polymerase incomplete primer extension (PIPE) [23] using the following primers, 5’‐GAACCTGTACTTCCAATCTGGACGTGGTAAGGGC‐3’ and 5’‐GTTATCCACTTCCAATGTTAACCGCCAAAGCC‐3’, and pEU_DmH4 [19] was used as a template for the histone H4 coding gene. The PIPE vector fragment was prepared by PCR reaction using the following primers, 5’‐CATTGGAAGTGGATAAC‐3’ and 5’‐TTGGAAGTACAGGTTC‐3’. Both PCR products were mixed and immediately transformed into JM109 competent cells (Takara Bio, Shiga, Japan), and transformants were sequence verified (Eurofins Genomics, Tokyo, Japan) by the same primer used to amplify the insert DNA.

### *In vitro* transcription

The plasmid harboring each histone or NAP1 was *in vitro* transcribed individually, as described previously [19]. All reagents used were purchased (CellFree Sciences, Yokohama, Japan). Briefly, 2 μg plasmid DNA was mixed with 20 U SP6 RNA polymerase, 20 U RNase inhibitor, 2.5 mm NTP mix, and 5× transcription buffer in a total reaction volume of 20 μL, and incubated for 4 h at 37 °C. The concentration of synthesized mRNA was quantified by UV spectrophotometry (DS11, DeNovix, Wilmington, DE, USA) after DNase I treatment for 30 min at 37 °C. Transcribed mRNAs were purified as previously described [19] and resuspended in RNase‐free water (Nippon Gene, Tokyo, Japan). Histone mRNAs were premixed before translation with a 1:1 ratio of H3:H4 for tetrasome assembly and a 2.5 : 2.5 : 1 : 1 ratio of H2A : H2B : H3 : H4 for nucleosome assembly.

### *In vitro* tetrasome and nucleosome assembly reactions

The tetrasome assembly reaction was performed by mixing 5 μL wheat germ cell‐free extract (WEPRO7240H Lot: 20CR02MN, CellFree Sciences), 4.4 μg H3/H4 mRNA mixture, 8.8 μg NAP1 mRNA, 0.4 μg creatine kinase, and 0.25 μg 601_185bp in a total of 10.7 μL reaction mixture. To form a diffusible bilayer reaction [24], the reaction mixture was carefully applied under the top layer consisting of 103 μL 1 × SUB‐AMIX. A co‐expression chromatin assembly reaction was performed for 6 h at 26 °C. For the nucleosome assembly reaction, 17.6 μg of the premixed four core histone mRNAs was used in the presence of either 0.25 μg 601_185 bp or 0.25 μg 601_307 bp sequence. The composition of reaction and protocol are otherwise the same as the tetrasome assembly reaction, described above. For AFM measurement, the nucleosome assembled on a 601_307 bp DNA fragment was used. Assembled tetrasomes and nucleosomes were incubated for 1 h at 55 °C to eliminate nonspecific histones‐DNA interactions [25].

### Affinity purification of assembled tetrasome and nucleosome

The assembled tetrasomes and nucleosomes were purified using a TALON Magnetic Beads (Takara Bio) via the His_6_‐tag designed at the N‐terminal of histone H4. Briefly, 50 μL TALON magnetic beads were rinsed with 300 μL deionized water and then equilibrated with 300 μL equilibration buffer (10 mm Tris/HCl pH7.4, 40 mm NaCl), and the supernatant was removed using a magnetic rack. 113.7 μL of the assembled sample was incubated with the beads for 1 h at 5 °C on a rotator. The supernatant was carefully removed, and the beads were washed 2 times with 500 μL wash buffer (10 mm Tris/HCl pH7.4, 40 mm NaCl, 10 mm Imidazole). Purified tetrasomes and nucleosomes were eluted in 25 μL elution buffer (10 mm Tris/HCl pH7.4, 40 mm NaCl, 250 mm Imidazole), in the presence of a trace amount of RNase A (Macherey‐Nagel GmbH & Co., Mannheim, Germany). The amount of reconstituted tetrasome and nucleosome before and after affinity purification was estimated by measuring nucleosomal and tetrasomal DNAs on a polyacrylamide gel by visual inspection.

### Restriction enzyme digestion of reconstituted tetrasomes

A 60 U *Pml*I (New England Biolabs, Beverly, MA, USA), 30 U *BsiW*I (New England Biolabs), or 30 U *Not*I (Takara Bio) was added to the 50 ng 601_185 bp or the purified tetrasome containing ˜ 15 ng DNA in an appropriate buffer system. The restriction enzyme digestion using *Pml*I and *Not*I was performed for 1 h at 37 °C, and *BsiW*I reaction was carried out for 1 h at 55 °C. After the reaction, the sample was deproteinized by phenol–chloroform‐IAA extraction and purified by ethanol precipitation, then resuspended in HD buffer (25 mm HEPES, 1 mm DTT, pH7.6) with trace amounts of RNase A and run on a 10% native polyacrylamide gel, followed by ethidium bromide staining. The band intensities were measured by the Gel‐Doc system (Bio‐Rad, Hercules, CA USA). The following equation calculated the %protection from *Pml*I and *BsiW*I digestions in the presence of tetrasome relative to naked DNA.

%protection=Remaining tetrasomal DNAIntactDNA‐RemainingnakedDNA×100%.

### Micrococcal nuclease assay

Purified nucleosomes were mixed with 0, 0.08, 0.4, or 1.6 U of Micrococcal nuclease (New England Biolabs) in the presence of 2.5 mm CaCl_2_. The reaction was incubated for 10 min at 37 °C, then halted by adding 5 mm EDTA, followed by phenol–chloroform‐IAA extraction and ethanol precipitation. Samples were analyzed by a 3% TBE agarose gel in a 1×TBE buffer system and visualized by ethidium bromide staining.

### Atomic force microscopy

The surface of mica (TED PELLA, INC, Redding, CA, USA) was treated with 1‐(3‐aminopropyl) silatrane (APS) as described previously [26] to immobilize the purified ˜ 0.5 nm tetrasome or nucleosome samples. Samples were placed on the mica for 5 min at room temperature, then rinsed with deionized water, and dried under nitrogen flow. AFM (NanoWizard III, JPK Instruments, Berlin, Germany) was used in tapping mode to scan the samples in the air with a silicon probe (OMCL‐AC160TS‐C3; Olympus, Tokyo, Japan). The scanning rate was 8.0 Hz over a scan area of 1 μm. The heights and diameters of tetrasomes (*n* = 100) or nucleosomes (*n* = 100) were measured using JPKSPM Data Processing software (JPK Instruments). The diameters of tetrasomes and nucleosomes were calculated by subtracting 2 × 7 nm (tip radius) from the observed diameters, accordingly to a manufacturer. Thresholds for tetrasomes and nucleosomes were made based on both height and diameter. For example, we considered the particles as tetrasomes when they meet criteria of both height and diameter (0.6 nm ≦ height ≦ 2.8 nm and 6 nm ≦ diameter ≦ 18 nm). Similarly, the particles satisfied 1.8 nm ≦height ≦ 3.4 nm and 12 nm ≦ diameter ≦ 24 nm were considered as nucleosomes.

## Results

### Tetrasome and nucleosome assembly using wheat germ co‐expression system

**Fig. 1:**
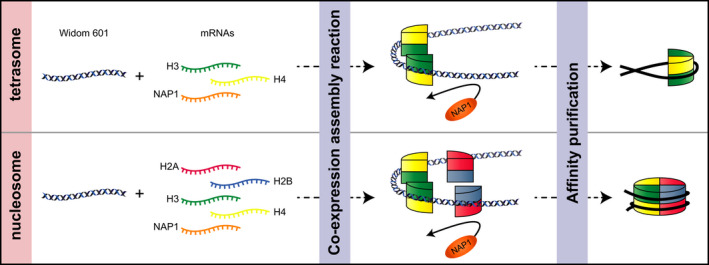
Schematic diagram of *in vitro* nucleosome and tetrasome assembly using a wheat germ protein co‐expression system. The mRNAs encoding *Drosophila* histone H2A, H2B, H3, and His_6_‐H4, and nucleosome assembly protein (NAP1) were co‐expressed in the presence of a short DNA fragment containing the Widom 601 sequence. Translational reaction and DNA‐histone assembly may occur simultaneously.

Previously, we reported a method to reconstitute chromatin by co‐expressing four core histones and chromatin assembly factors using a wheat germ cell‐free system [19]. To improve this technology for more applicable protocols in the area of chromatin research, we aimed to assemble *Drosophila* monotetrasome and mononucleosome on a well‐described Widom 601 sequence followed by affinity purification using N‐terminal His_6_ tag on histone H4 accordingly to manufacturer's instructions [27]. A flowchart of this study from a preparation of mRNAs, co‐expression/histone‐DNA complex assembly reactions, followed by purification is shown in Fig. 1. A pair of H3 and H4‐coding mRNAs, or a set of four core histone‐coding mRNAs is used together with NAP1‐coding mRNA, and they were co‐expressed in the presence of a short double‐stranded DNA possessing the Widom 601 sequences at the center.

### Characterization of assembled tetrasomes

**Fig. 2:**
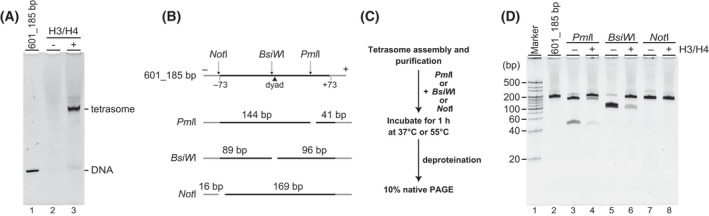
Analysis of assembled tetrasomes. (A) Reconstituted and purified tetrasomes were analyzed by 6% native polyacrylamide gels and visualized with ethidium bromide. Lane 1 indicates the naked 601_185 bp DNA. Lanes 2 and 3 show the formation of tetrasomes in the absence or presence of H3/H4 mRNAs in the co‐expression assembly reaction followed by a His_6_‐tag purification, respectively. (B) The cleavage sites of restriction enzymes, *Not*I, *BsiW*I, and *Pml*I on 601_185 bp are indicated. The filled triangle indicates the dyad of the Widom 601 sequence. (C) Scheme of restriction enzymes assays on the purified tetrasomes, in which *Pml*I and *Not*I were reacted at 37 °C, and *BsiW*I was incubated at 55 °C. (D) DNA fragments after restriction enzyme digestions were analyzed on 10% native PAGE. 601_185 bp derived from the purified tetrasomes in the absence of restriction enzyme is shown (lane 1). Naked 601_185 bp or tetrasome, indicated as ± H3/H4, was digested with *Pml*I,, *BsiW*I, and *Not*I.

First, we tested whether a tetrasome can be assembled by our method. A pair of H3 and H4 mRNAs was co‐expressed with NAP1 mRNA in the presence of 601_185 bp for 6 h at 26 °C, followed by affinity purification. In the absence of H3/H4 mRNAs, neither mobility shift nor free DNA was determined by electrophoretic mobility shift assay (EMSA) as was expected (Fig. 2A). In contrast, a tetrasomal DNA was found by EMSA when a complete tetrasome reconstitution and following purification were performed. The amount of reconstituted tetrasome before and after purification was estimated to be 145.4 ± 21.1 ng per reaction (*n* = 3) and 17.6 ± 0.7 ng per reaction (*n* = 3), respectively. Thus, ˜ 12% of tetrasome was purified by affinity purification. By considering the migration of tetrasomes on the gel, it was suggested that only one tetrasome is formed on a given sequence. To address the position of the assembled tetrasome on a 601_185 bp, the following restriction enzymes, *PmI*l, *BsiW*I, and *Not*I, were used for the digestion of assembled and purified tetrasomes (Fig. 2B). The cleavage sites of each restriction enzyme are shown in Fig. 2B, so that detected length from the end products should give us an idea of where roughly the tetrasome is positioned on the 601_185 bp. Tetrasomes were digested by each restriction enzyme, and then, end products were analyzed on a 10% native polyacrylamide gel (Fig. 2C and D). The digestion either by *Pml*I or *BsiW*I on a tetrasome was significantly reduced (Fig. 2D, lanes 4, and 6), compared to that of naked DNAs (Fig. 2D, lines 3 and 5). The %protections from *Pml*I and *BsiW*I by a tetrasome on 601_185 bp were estimated to be 81.0 ± 6.3% (*n* = 3) and 93.6 ± 4.2% (*n* = 3), respectively. In contrast, when either naked or tetrasome assembled 601_185 bp was digested by *Not*I, both samples were completely digested (Fig. 2D lane 7‐8). We concluded that the majority of tetrasomes was assembled near the center of 601 flanked by *Pml*I and *BsiW*I recognition sites (Fig. 2B).

### Assessment of reconstituted nucleosomes

**Fig. 3:**
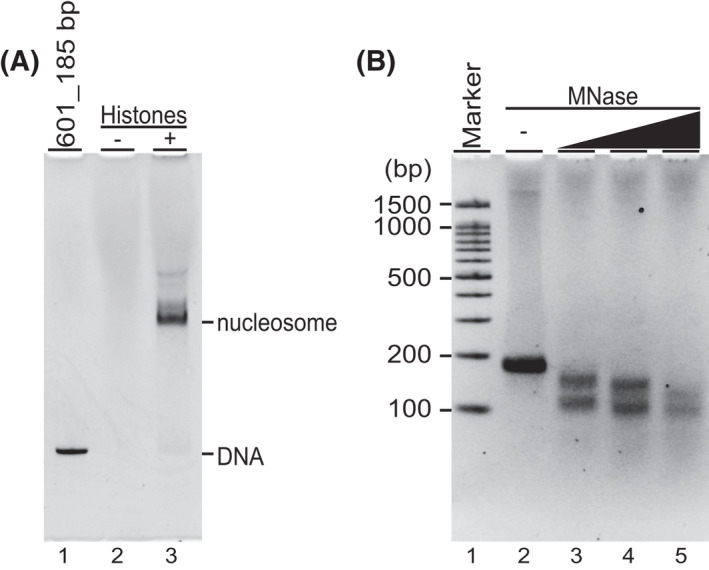
Reconstituted nucleosomes. (A) Assembled and purified nucleosomes were assessed by 6% native PAGE. Lanes 1‐3 indicate the naked 601_185 bp, the reactions in the absence or presence of histone mRNAs in a co‐expression reconstitution reaction, respectively. (B) Purified nucleosomes were digested by increasing amounts of micrococcal nuclease, 0, 0.08, 0.4, 1.6 U for 10 min at 37 °C. The samples were analyzed by a 3% agarose TBE gel and stained with ethidium bromide.

Next, we analyzed the assembled then purified nucleosomes, consisting of histones, H2A, H2B, H3, and H4. Four core histones and NAP1‐coding mRNAs in the presence of 601_185 bp were used in a co‐expression reaction for 6 h at 26 °C, followed by affinity purification. In the absence of core histone‐coding mRNAs, no nucleosome nor DNA was detected (Fig. 3A, line 2). In the presence of all necessary components, the reconstituted nucleosome was determined (Fig. 3A, lane 3). The amount of reconstituted nucleosome prior to and after purification was estimated to be 201.2 ± 13.1 ng per reaction (*n* = 3) and 19.6 ± 1.6 ng per reaction (*n* = 3), respectively. Thus, ˜ 10% of tetrasome was purified by affinity purification. Thus, the core histones‐DNA complexes were formed in high purity. To assess if the nucleosomes had a canonical structure, that is, a DNA wrapping 1.7 times around core histones, a MNase assay was performed (Fig. 3B). After 10 min of MNase digestion, about 153 bp DNA fragment was observed (Fig 3B, lanes 3 and 4), consistent with the reported core nucleosomal DNA length [2, 28]. When an excess amount of MNase was added to the nucleosome, most of the DNA wrapping around histone octamer was diminished (Fig 3B, lane 5). Additionally, around 100‐110 bp subnucleosomal DNA fragment was found after MNase digestion. Observed subnucleosomal DNA fragments from MNase digestion will be discussed later. From these results, we concluded that the purified nucleosome maintained an expected geometry.

### Atomic force microscopy analysis of purified tetrasomes and nucleosomes

**Fig. 4:**
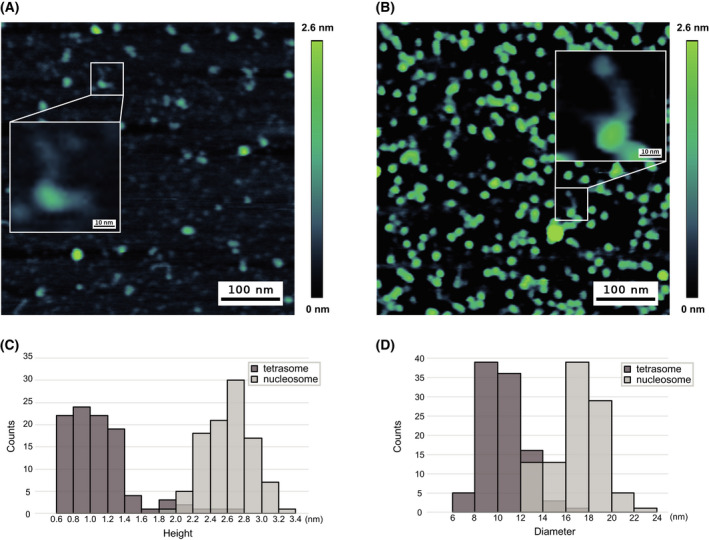
Atomic force microscope analysis of reconstituted tetrasomes and nucleosomes. Representative images of a purified tetrasome on 601_185bp (A), and nucleosome on 601_307 bp (B) are shown (scale bar = 100 nm), respectively. (C) The distribution of heights of 100 each tetrasomes and nucleosomes are shown. (D) The distribution of diameters of 100 each tetrasomes and nucleosomes are shown.

Purified tetrasomes on 601_185 bp and nucleosomes on 601_307 bp (80bp non‐nucleosome positioning sequences flank the Widom 601 sequence) were placed on an APS coated mica, and the topography of the samples was analyzed by AFM. To note, the reconstituted nucleosomes on 601_307 bp were purified and confirmed (Fig. S1). This construct was expected to add two ˜ 80 bp free DNA ends from the nucleosome core particle under AFM measurement. Typical images of the tetrasome on 601_185 bp and nucleosome on 601_307 bp are shown in Fig. 4A and B (Fig. S2), respectively. From 100 each tetrasomes and nucleosomes, distributions of heights were analyzed (Fig. 4C). The average and standard deviation of the tetrasomes were estimated to be 1.2 ± 0.4 nm (*n* = 100), statistically significantly different from that of the nucleosomes with 2.7 ± 0.3 nm (*n* = 100), *P*‐value <0.001 (*t*‐test). The diameters of the tetrasomes and nucleosomes were also distinguishable (*P*‐value <0.001, *t*‐test), 10.1 ± 1.8 nm and 16.5 ± 2.3 nm, respectively (Fig. 4D).

## Discussion

Here, we reported a method to prepare purified tetrasomes and nucleosomes on the Widom 601 sequence under a NAP1‐mediated co‐expression assembly reaction, which could provide a preferable substrate for epigenetic enzyme screening (Fig. 1). The function of NAP1 during a nucleosome assembly is suggested to prevent non‐nucleosomal histone‐DNA interactions [9, 29, 30]. Consistent with the previously reported chaperone‐mediated tetrasome assembly using recombinant histones H3 and H4, we were able to assemble tetrasomes under a novel co‐expression mode (Fig. 2A). It was suggested that there is only one tetrasome formed per short DNA sequence, and we assessed the position of tetrasomes on the 601_185 bp sequence using restriction enzymes (Fig. 2B‐D). *Not*I, *BsiW*I, and *Pml*I recognition sites are located at −75 to −82, −3 to −8, and +49 to +54, relative to a dyad, respectively, and the region between −8 and +54 was indicated to be protected by a positioned tetrasome. In an earlier study, it was shown that the tetramer is the major determinant of the nucleosome positioning on the 208 bp sea urchin 5S rRNA sequence [31]. More recently, a sequence‐dependent tetrasome positioning on the Widom 601 sequence was shown to be due to the high bendability and twistability of this sequence, which in turn stabilize the histone‐DNA complex [30]. Thus, the reconstituted tetrasomes in this study using co‐expression assembly are thought to be comparable to the tetrasomes assembled by salt gradient methods.

The purified reconstituted nucleosomes using NAP1‐mediated co‐expression reconstitution on 601_185 bp were also confirmed (Fig. 3A), and MNase assay was performed to determine the length of nucleosomal DNA (Fig. 3B). Around 150 bp DNA fragment was produced by a partial MNase digestion, suggesting that a canonical nucleosome was formed. Additionally, observed 100‐110 bp fragments are thought to be derived from subnucleosomal species, which might be produced during the MNase digestion (Fig. 3A). It is known that the nucleosome core particle can be invaded by the aggressive nuclease activity of MNase, resulting in shorter DNA fragments [32]. Hence, observed 100‐110 bp fragments were thought to be derived from the DNA wrapping around H2A/H2B/(H3/H4)_2_ hexamer, consistent with reported notions [33, 34, 35].

Finally, purified tetrasomes and nucleosomes were analyzed by AFM, and the heights and diameters were measured (*n* = 100 each) (Fig. 4 and Fig. S2). The tetrasome on a 601_185 bp shown in Fig. 4A indicated that there are ˜ 20 and ˜ 14 nm free DNAs sticking out from the core particle to the direction toward 12‐ and 3‐o'clock, respectively, which correspond to the expected free DNA length (bp) of ˜ 60 and ˜ 40 bp, respectively. Similarly, observed ˜ 33 and ˜ 22 nm free DNAs sticking out from the core particle to the 12‐ and 4‐o'clock directions, respectively, are in accordance with the expected ˜ 98 and ˜ 65 bp of non‐nucleosomal DNA on a 601_307 bp construct (Fig. 4B). However, the DNAs sticking out from the tetrasomes and nucleosomes were only seen in limited number of samples due to lack of resolution. The heights and diameters between the tetrasomes and nucleosomes were statistically different from each other (Fig. 4C). In agreement with the previous studies, the average height and diameter of nucleosomes measured in this study are around 2.7 and 16.5 nm, respectively (Fig. 4C and D) [36, 37]. Moreover, the average diameter of the tetrasomes of around 10 nm is consistent with the previous report [37]. Of note, we were not able to compare the height of the tetrasomes measured in this study to others due to limited data describing the height of the tetrasomes acquired from the AFM image. Alternatively, we estimated the volume of observed tetrasomes in our analysis accordingly to the previous study [38]. The volume of reconstituted tetrasomes in our analysis was 49 ± 28 nm^3^ (*n* = 100), which is well within the range of the reported volumes of tetrasomes obtained by the molecular dynamics simulation [35].

In summary, we successfully reconstituted both tetrasomes and nucleosomes by using a wheat germ co‐expression system in the presence of the Widom 601 sequence, followed by affinity purification. Both reconstituted tetrasomes and nucleosomes seemed stably positioned on the nucleosome positioning sequence; hence, they were successfully purified. The purified tetrasomes and nucleosomes using our method may allow us to understand the mechanisms of chromatin assembly and disassembly dynamics by evaluating the functions of chromatin assembly factors under near physiological condition. Furthermore, purified tetrasome and nucleosome should be favorable nonmodified homogeneous substrates for downstream applications such as epigenetic enzyme screening [20]. In such case, a TEV protease can be used during affinity purification process, which will provide a tag‐free N‐terminal histone H4‐containing tetrasomes or nucleosomes [22]. Overall, our current method has clear advantages over classical reconstitution methods [20] (i.e., assembled tetrasomes and nucleosomes in a co‐expression reaction are in native state and they are free from typical epigenetic modifications). However, it should be stated here that there are several limitations. For example, the product yield is not comparable to the conventional methods, which utilize recombinantly expressed histones, unless we increase the reaction volume of the current protocol more than 100 times. Additionally, we do not know the atomic‐level structure of our reconstituted tetrasomes and nucleosomes to date, so a large‐scale reconstitution and subsequent structural determination such as X‐ray crystallography or Cryo‐EM are needed in future research.

## Conflict of interest

The authors declare that they have no known competing financial interests or personal relationship that could have appeared to influence the work reported in this paper.

## Data accessibility

The data that support the findings of this study are available from the corresponding author (takasuka@cen.agr.hokudai.ac.jp) upon reasonable request.

## Author contributions

KO and TET designed the research. KO, SH, KM, and KU performed experiments. KO, PB, ST, and TET wrote the manuscript.

## Supporting information


**Fig. S1**. Purified reconstituted nucleosome on 601_307bp, analyzed by 6% PAGE.

**Fig. S2**. Overview of AFM images shown in Fig. 2. Tetrasome (A) and nucleosome (B) particles are shown with color‐coded height (0–2.6 nm) (scale bar = 100 nm).

## References

[1] Kornberg RD . Structure of chromatin. Annu Rev Biochem. 1977;46:931–54. 332067 10.1146/annurev.bi.46.070177.004435

[2] Luger K , Mäder AW , Richmond RK , Sargent DF , Richmond TJ . Crystal structure of the nucleosome core particle at 2.8 Å resolution. Nature. 1997;389:251–60. 9305837 10.1038/38444

[3] Kornberg RD , Lorch Y . Twenty‐five years of the nucleosome, fundamental particle of the eukaryote chromosome. Cell. 1999;98:285–94. 10458604 10.1016/s0092-8674(00)81958-3

[4] Shibahara KI , Stillman B . Replication‐dependent marking of DNA by PCNA facilitates CAF‐1‐coupled inheritance of chromatin. Cell. 1999;96:575–85. 10052459 10.1016/s0092-8674(00)80661-3

[5] Rice JC , Allis CD . Lysine methylation and acetylation of histones. Curr Opin Cell Biol. 2001;13:263–73. 11343896 10.1016/s0955-0674(00)00208-8

[6] Zhu B , Chen S , Wang H , Yin C , Han C , Peng C , et al. The protective role of DOT1L in UV‐induced melanomagenesis. Nat Commun. 2018;9:259. 29343685 10.1038/s41467-017-02687-7 PMC5772495

[7] De Koning L , Corpet A , Haber JE , Almouzni G . Histone chaperones: an escort network regulating histone traffic. Nat Struct Mol Biol. 2007;14:997–1007. 17984962 10.1038/nsmb1318

[8] Akey CW , Luger K . Histone chaperones and nucleosome assembly. Curr Opin Struct Biol. 2003;13:6–14. 12581654 10.1016/s0959-440x(03)00002-2

[9] Andrews AJ , Chen X , Zevin A , Stargell LA , Luger K . The histone chaperone Nap1 promotes nucleosome assembly by eliminating nonnucleosomal histone DNA interactions. Mol Cell. 2010;37:834–42. 20347425 10.1016/j.molcel.2010.01.037 PMC2880918

[10] Torigoe SE , Patel A , Khuong MT , Bowman GD , Kadonaga JT . ATP‐dependent chromatin assembly is functionally distinct from chromatin remodeling. Elife. 2013;2:e00863. 23986862 10.7554/eLife.00863 PMC3748710

[11] Böhm V , Hieb AR , Andrews AJ , Gansen A , Rocker A , Tóth K , et al. Nucleosome accessibility governed by the dimer/tetramer interface. Nucleic Acids Res. 2011;39:3093–102. 21177647 10.1093/nar/gkq1279 PMC3082900

[12] Biochem J , Ishimi YF , Hirosumi MJ , Sciences Y , Chemistry P . Structures which In Facilitates Assembly of Nucleosome‐Like Cells ’ Vitro in Mammalian Chemistry, Faculty of Pharmaceutical detected When these In eukaryotic cells, chromatin consists of a repeat‐ ing unit called the nucleosome (1, 2). To know the Me. J Biochem. 1983;94:735–44.6643419

[13] Ito T , Bulger M , Kobayashi R , Kadonaga JT . Drosophila NAP‐1 is a core histone chaperone that functions in ATP‐facilitated assembly of regularly spaced nucleosomal arrays. Mol Cell Biol. 1996;16:3112–24. 8649423 10.1128/mcb.16.6.3112 PMC231306

[14] Mosammaparast N , Jackson KR , Guo Y , Brame CJ , Shabanowitz J , Hunt DF , et al. Nuclear import of histone H2A and H2B is mediated by a network of karyopherins. J Cell Biol. 2001;153:251–62. 11309407 10.1083/jcb.153.2.251 PMC2169462

[15] Mazurkiewicz J , Kepert JF , Rippe K . On the mechanism of nucleosome assembly by histone chaperone NAP1. J Biol Chem. 2006;281:16462–72. 16531623 10.1074/jbc.M511619200

[16] Zlatanova J , Seebart C , Tomschik M . Nap1: taking a closer look at a juggler protein of extraordinary skills. FASEB J. 2007;21:1294–310. 17317729 10.1096/fj.06-7199rev

[17] Tripathi AK , Singh K , Pareek A , Singla‐Pareek SL . Histone chaperones in Arabidopsis and rice: Genome‐wide identification, phylogeny, architecture and transcriptional regulation. BMC Plant Biol. 2015;15:1–25. 25849155 10.1186/s12870-015-0414-8 PMC4357127

[18] Andrews AJ , Downing G , Brown K , Park YJ , Luger K . A thermodynamic model for Nap1‐histone interactions. J Biol Chem. 2008;283:32412–8. 18728017 10.1074/jbc.M805918200 PMC2583301

[19] Kei‐ichi O , Nagy SK , Hataya S , Endo Y , Takasuka TE . Reconstitution of Drosophila and human chromatins by wheat germ cell‐free co‐expression system. BMC Biotechnol. 2020;20:1–12. 33261588 10.1186/s12896-020-00655-6 PMC7708258

[20] Endo Y , Takemori N , Nagy SK , Okimune K‐I , Kamakaka R , Onouchi H , et al. De novo reconstitution of chromatin using wheat germ cell‐free protein synthesis. FEBS Open Bio. 2021;11:1552. 10.1002/2211-5463.13178 PMC8167859 33960726

[21] Khuong MT , Fei J , Cruz‐Becerra G , Kadonaga JT . A simple and versatile system for the ATP‐dependent assembly of chromatin. J Biol Chem. 2017;292:19478–90. 28982979 10.1074/jbc.M117.815365 PMC5702684

[22] Blommel PG , Fox BG . A combined approach to improving large‐scale production of tobacco etch virus protease. Protein Expr Purif. 2007;55:53–68. 17543538 10.1016/j.pep.2007.04.013 PMC2047602

[23] Klock HE , Koesema EJ , Knuth MW , Lesley SA . Combining the polymerase incomplete primer extension method for cloning and mutagenesis with microscreening to accelerate structural genomics efforts. Proteins. 2008;71:982–94. 18004753 10.1002/prot.21786

[24] Takai K , Sawasaki T , Endo Y . Practical cell‐free protein synthesis system using purified wheat embryos. Nat Protoc. 2010;5:227–38. 20134421 10.1038/nprot.2009.207

[25] Tachiwana H , Kagawa W , Osakabe A , Kawaguchi K , Shiga T , Hayashi‐Takanaka Y , et al. Structural basis of instability of the nucleosome containing a testis‐specific histone variant, human H3T. Proc Natl Acad Sci USA. 2010;107:10454–9. 20498094 10.1073/pnas.1003064107 PMC2890842

[26] Lyubchenko YL , Gall AA , Shlyakhtenko LS . Visualization of DNA and protein‐DNA complexes with atomic force microscopy. Methods Mol Biol. 2014;1117:367–84. 24357372 10.1007/978-1-62703-776-1_17 PMC4226758

[27] Lowary PT , Widom J . New DNA sequence rules for high affinity binding to histone octamer and sequence‐directed nucleosome positioning. J Mol Biol. 1998;276:19–42. 9514715 10.1006/jmbi.1997.1494

[28] Davey CA , Sargent DF , Luger K , Maeder AW , Richmond TJ . Solvent mediated interactions in the structure of the nucleosome core particle at 1.9 Å resolution. J Mol Biol. 2002;319:1097–113. 12079350 10.1016/S0022-2836(02)00386-8

[29] Vlijm R , Lee M , Lipfert J , Lusser A , Dekker C , Dekker NH . Nucleosome assembly dynamics involve spontaneous fluctuations in the handedness of tetrasomes. Cell Rep. 2015;10:216–25. 25578730 10.1016/j.celrep.2014.12.022

[30] Ordu O , Lusser A , Dekker NH . DNA sequence is a major determinant of tetrasome dynamics. Biophys J. 2019;117:2217–27. 31521330 10.1016/j.bpj.2019.07.055 PMC6895708

[31] Dong F , Van Holde KE . Nucleosome positioning is determined by the (H3–H4)2 tetramer. Proc Natl Acad Sci USA. 1991;88:10596–600. 1961726 10.1073/pnas.88.23.10596 PMC52976

[32] Chereji RV , Bryson TD , Henikoff S . Quantitative MNase‐seq accurately maps nucleosome occupancy levels. Genome Biol. 2019;20:198. 31519205 10.1186/s13059-019-1815-z PMC6743174

[33] Teves SS , Henikoff S . Heat shock reduces stalled RNA polymerase II and nucleosome turnover genome‐wide. Genes Dev. 2011;25:2387–97. 22085965 10.1101/gad.177675.111 PMC3222904

[34] Arimura Y , Tachiwana H , Oda T , Sato M , Kurumizaka H . Structural analysis of the hexasome, lacking one histone H2A/H2B dimer from the conventional nucleosome. Biochemistry. 2012;51:3302–9. 22448809 10.1021/bi300129b

[35] Rychkov GN , Ilatovskiy AV , Nazarov IB , Shvetsov AV , Lebedev DV , Konev AY , et al. Partially assembled nucleosome structures at atomic detail. Biophys J. 2017;112:460–72. 28038734 10.1016/j.bpj.2016.10.041 PMC5300784

[36] Wang H , Bash R , Lindsay SM , Lohr D . Solution AFM studies of human Swi‐Snf and its interactions with MMTV DNA and chromatin. Biophys J. 2005;89:3386–98. 16100261 10.1529/biophysj.105.065391 PMC1366835

[37] Zou T , Hashiya F , Wei Y , Yu Z , Pandian GN , Sugiyama H . Direct observation of H3–H4 octasome by high‐speed AFM. Chem ‐ A Eur J. 2018;24:15998–6002. 10.1002/chem.201804010 30088306

[38] Nazarov I , Chekliarova I , Rychkov G , Ilatovskiy AV , Crane‐Robinson C , Tomilin A . AFM studies in diverse ionic environments of nucleosomes reconstituted on the 601 positioning sequence. Biochimie. 2016;121:5–12. 26586109 10.1016/j.biochi.2015.11.010

