# Supplementary material for: Histone chaperone‐mediated co‐expression assembly of tetrasomes and nucleosomes
Source: FEBS Open Bio. 2021 Oct 19;11(11):2912–20. doi: 10.1002/2211-5463.13311 (PMC8564334; doi:10.1002/2211-5463.13311)
Supplement: Supplementary file 1 — Fig. S1. Purified reconstituted nucleosome on 601_307bp, analyzed by 6% PAGE. [file FEB4-11-2912-s001.pdf]

Supplementary Figure 1

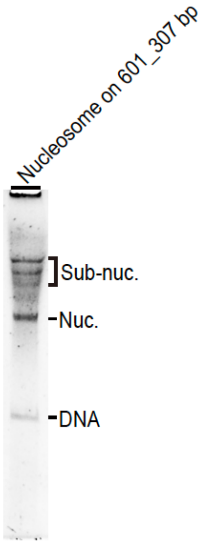

Supplementary Figure 1. Purified reconstituted nucleosome on 601_307bp, analyzed by 6% PAGE.
