# Supplementary material for: Histone chaperone‐mediated co‐expression assembly of tetrasomes and nucleosomes
Source: FEBS Open Bio. 2021 Oct 19;11(11):2912–20. doi: 10.1002/2211-5463.13311 (PMC8564334; doi:10.1002/2211-5463.13311)
Supplement: Supplementary file 2 — Fig. S2. Overview of AFM images shown in Fig. 2. Tetrasome (A) and nucleosome (B) particles are shown with color‐coded height (0–2.6 nm) (scale bar = 100 nm). [file FEB4-11-2912-s002.docx]

Supplementary Figure 2

**Supplementary Figure 2.** Overview of AFM images shown in Fig. 2. Tetrasome (A) and nucleosome (B) particles are shown with color-coded height (0 - 2.6 nm).
